# Assessing the risk factors associated with sarcopenia in patients with liver cirrhosis: a case–control study

**DOI:** 10.1038/s41598-023-48955-z

**Published:** 2023-12-09

**Authors:** LeYao Xiao, Mei Dai, Fei Zhao, YouShu Shen, Rick Yiu Cho KWAN, Jordan Tovera Salvador, Li Zhang, YaWen Luo, Qian Liu, Ping Yang

**Affiliations:** 1 Deparment of Infectious Diseases, Affiliated Hospital of Zunyi Medical University, Zunyi, 563000 China; 2 School of Nursing, Zunyi Medical University, Zunyi, 563000 China; 3 School of Nursing, Tung Wah College, Hong Kong SAR, China; 4 Nursing Education Department, College of Nursing, Imam Abdulrahman Bin Faisal University, Dammam, Saudi Arabia; 5 Nursing Department of the Affiliated Hospital of Zunyi Medical University, Zunyi, China; 6 Philippine Women’s University, Manila, Philippines

**Keywords:** Nutrition disorders, Risk factors

## Abstract

Sarcopenia is a disease characterized by decreased muscle mass and strength, affecting 20–70% of patients with cirrhosis, and is associated with poor prognosis, complications, and high mortality. At present, the epidemiological investigation of sarcopenia in patients with liver cirrhosis is relatively limited, and because of the differences in population characteristics, regions, diagnostic criteria and diagnostic tools, the prevalence of sarcopenia in various studies varies greatly. The definition of sarcopenia in this study adopted the criteria of the Asian Working Group on Sarcopenia (AWGS 2019), including muscle mass and muscle strength / physical performance. A total of 271 patients with liver cirrhosis were included in this cross-sectional study to explore the influencing factors of sarcopenia in patients with liver cirrhosis. The prevalence of sarcopenia was 27.7%, 27.3% in male and 28.4% in female. The results of binary logistic regression analysis showed that age, physical activity, BMI, mid-upper arm muscle circumference, hepatic encephalopathy, nutritional status, alkaline phosphatase, albumin and total cholesterol were significantly correlated with the occurrence of sarcopenia in patients with liver cirrhosis. After adjusting for the potential influencing factors, it was found that the correlation between age and sarcopenia was weakened (OR = 0.870, 95% CI 0.338–2.239). The current findings show that sarcopenia is common in patients with cirrhosis and is independently associated with age, physical activity, BMI, nutritional status, and albumin, and serum alkaline phosphatase and total cholesterol are associated with the development of sarcopenia. Regular exercise may help maintain the grip strength of patients with cirrhosis and delay the deterioration of liver function.

## Introduction

Sarcopenia is considered to be a progressive systemic skeletal muscle disorder associated with an increased likelihood of adverse outcomes such as falling down, bone fracture, physical disability, and death^1^. The 2016 World Health Organization International Classification of Diseases code for sarcopenia, ICD-10, has important clinical implications for the study of this disease^2^. With the widespread clinical recognition of muscle loss in chronic disease, aging-related muscle loss is considered “primary sarcopenia,” while "secondary sarcopenia" is sarcopenia occurring in chronic diseases^3^ such as advanced liver, heart, lung, and kidney diseases. Both primary and secondary sarcopenia are states of anabolic resistance or attenuated muscle response to nutrition and physical activity^4^. Among the various diseases related to secondary sarcopenia, the incidence of sarcopenia among patients with cirrhosis is high (20–70%)^5^, significantly higher than the rates observed in the general older people population (2.9–38.5%)^6,7^.

Studies have revealed sarcopenia is a significant predictor of mortality both before and after liver transplantation^8^. The causes of sarcopenia in liver cirrhosis (LC) are complex and multifactorial, encompassing factors such as inadequate energy intake, an accelerated starvation response, the liver-muscle axis, and systemic inflammation. Furthermore, sarcopenia is associated with an increased risk of infection, prolonged hospital stays, hepatic encephalopathy, poor quality of life, and increased healthcare costs among patients with cirrhosis^8,9^. Several studies have reported that exercise training and nutritional interventions can slow down the progression or even reverse muscle wasting, leading to improvements in physical function and frailty in patients with cirrhosis^10^. Several studies with small number of cases enrolled suggested that sarcopenia in liver cirrhotic patients might associated with the severity, etiology of liver disease, malnutrition, or alcohol intake^11^. However, the risk factors associated with sarcopenia in patients with liver cirrhosis has not been adequately assessed.

Therefore, this study aims to investigate the risk factors associated with sarcopenia in patients with liver cirrhosis, enabling the identification of high-risk groups and the development of early preventive interventions for sarcopenia in patients with cirrhosis.

## Materials and methods

### Study design

This study employed a case–control, observational, and descriptive exploratory design and followed the Strengthening the Reporting of Observational Studies in Epidemiology (STROBE) guidelines^12^.

### Setting

Study participants were cirrhotic patients hospitalized in the Department of Infection and Gastroenterology at the Affiliated Hospital of Zunyi Medical University from July 2021 to July 2022. The Department of Infection serves approximately 500 patients with LC annually, while the Gastroenterology Department serves nearly 1000 such patients. The hospital is a tertiary teaching hospital and a leading medical center in the province.

### Participants

Participants were recruited based on the following eligibility criteria, and all data for the eligibility screening were obtained from medical records in the hospital.

#### Inclusion criteria

- Adult, age ≥ 18 years; and
- Diagnosis of cirrhosis based on typical clinical presentation, laboratory tests, imaging features, and/or representative pathological findings from liver biopsies.

#### Exclusion criteria

- Comorbidity with other serious systemic diseases or presence of other end-stage complications (e.g., chronic obstructive pulmonary disease, renal failure, heart failure, and other malignancies);
- Pregnant or lactating women;
- Diseases that may lead to impaired nutritional metabolism, including inflammatory bowel disease and thyroid disease; and.
- Receiving treatments with glucocorticoids or immunosuppressive drugs.

The study was approved by the Medical Ethics Committee of the Affiliated Hospital of Zunyi Medical University (reference number: KLLY-2021-149). Written informed consent was obtained from all participants. All methods were carried out in accordance with relevant guidelines and regulations of the Affiliated Hospital of Zunyi Medical University Ethics Committee.

### Variables and measurement

#### Outcome

Sarcopenia was assessed as a dichotomous variable. According to the Asian Working Group for Sarcopenia 2019 (AWGS 2019), the diagnostic indicators of sarcopenia include three aspects: SMI, muscle strength, and physical mobility). The diagnosis of sarcopenia is made when the first criterion, plus the second and/or third criteria, are met^13^.

#### SMI

CT images were analyzed by two independent radiologists using the Slice-O-matic V5.0 software. Muscle tissues on CT images were based on Hounsfield unit (HU) thresholds, ranging from -29 HU to + 150 HU^14^. Skeletal muscle area (SMA) was measured by scanning the third lumbar spine vertebra cross-section, and the L3-SMI was calculated as the ratio of SMA to squared height (cm^2^/m^2^). The cut-off point for low SMI was defined as SMI ≤ 52.4 cm^2^/m^2^ for men and SMI ≤ 38.5 cm^2^/m^2^ for women^15^.

#### Muscle strength

Hand grip strength (HGS) measurements were performed using an electronic hand grip strength device (EH 101, from Xiangshan, Guangdong, China) following the standardized grip strength measurement guidelines established by the American Manual Therapy Association^16^. Each participant was tested by maintaining a right angle (90°) to both hands, twice for both the right and left hands, using the average of the available maximum strength data. In cases where measuring one hand was not possible, the maximum value for the other hand was recorded. The cut-off point for low grip strength was defined as < 28 kg for men and < 18 kg for women^13^.

#### Physical mobility

The 6-m walk test was used^17^. Participants were instructed to walk at their usual walking speed for six meters. A gait speed of < 1.0 m/s was diagnosed as poor physical mobility^13^.

### Risk factors

The demographic data included gender, age, ethnicity, marital status, nature of work, educational attainment, residential area, co-living status, smoking, drinking, physical activity (≥ 30 min/time), monthly income, and medical insurance status.

The clinical data included the etiology of cirrhosis, duration of cirrhosis, stage of cirrhosis, hospitalization, cirrhosis-related complications, comorbidities, and prognosis based on Child–Pugh classification.

The cirrhosis-related biochemical data included hemoglobin, clotting profile, lipid profile, and liver function. The anthropometric data included height, weight, body mass index (BMI), triceps skinfold thickness (TSF), mid-upper arm muscle circumference, and waist-to-hip ratio.

The nutrition status was measured using the Royal Free Hospital-Nutritional Prioritizing Tool (RFH-NPT), which considers factors such as alcoholic hepatitis, tube feeding, body fluid retention status, BMI, unplanned body mass loss, and dietary intake reduction^18^. The scores obtained were used to classify the nutritional risk levels, with 0 indicating low risk of malnutrition, 1 for moderate risk, and 2–7 for high risk. This tool enables the classification of the nutritional risk of patients into three levels within 3 min: low, medium, and high. At present, this tool has been widely used in assessing the nutritional risk of patients, with research indicating its superior effectiveness in patients with LC compared to most clinical nutritional evaluation tools^18^.

### Confounding factors

Given the study aimed to identify risk factors, confounders could not be pre-identified without the risk factors known. This study considered only old age (i.e., age ≥ 60 years) and gender as confounders.

### Assessments of sarcopenia risk

The assessment of sarcopenia in this study was performed according to the diagnostic criteria for sarcopenia recommended by the Asian Working Group on Sarcopenia (AWGS 2019), when patients had low muscle mass and the presence of low muscle strength and / or low physical performance (Table 1).

**Table 1:** Diagnostic criteria of sarcopenia.

Diagnostic criteria of 2019 AWGS	Diagnostic indicators	Cut-off points	
		Male	Female
Low muscle mass	ASM adjusted for height^2^	≤ 7.0 kg/m^2^	≤ 5.7 kg/m^2^
Low muscle strength	Muscle strength	< 28 kg	< 18 kg
Low physical performance	6-m walk test	< 1 m/s	< 1 m/s

### Study size

The sample size was calculated using the formula: N = 4 (${\mathrm{U}}_{\mathrm{\alpha }}$ S/$\updelta$)^2^, where U is the test level, ${\mathrm{U}}_{\mathrm{\alpha }}$ represents the $\mathrm{\alpha }$ corresponding U value, S represents the standard deviation, δ represents the tolerance error, and $\mathrm{\alpha }$=0.05. In this study, a two-sided test was performed with U = 1.96 and δ = [0.25S, 0.5S]^5^. Considering the possible existence of invalid samples or data, the study increased the sample size by 20%. The calculated sample size range was approximately 75–296 cases, and 271 patients were finally included in this study.

### Statistical analysis

Before any statistical comparison, the Kolmogorov–Smirnov test and homogeneity variance test were used to test the normality of standard normal distribution for all variables. In descriptive analysis, continuous variables were described with mean and standard deviation or median and interquartile range, whereas categorical variables were described using frequency and percentage. To indicate the association between independent risk factors and sarcopenia, the Student’s t-test was used for continuous variables, and the χ^2^ test was used for categorical variables.

The logistic regression model included the following covariates: age, physical activity, BMI, mid-upper arm muscle circumference, hepatic encephalopathy, nutritional status, alkaline phosphatase, albumin, and total cholesterol, and considered gender as a potential sarcopenia related factor. A three-step approach was employed to identify the risk factors associated with sarcopenia. First, risk factors for sarcopenia were evaluated and identified using simple regression analysis (i.e., Model 1). Second, adjusted binary regression was performed using a stepwise method. Three models were used to illustrate the association between these risk factors and sarcopenia: Model 1 represents the unadjusted model, Model 2 incorporates all significant risk factors identified using the stepwise method, and Model 3 represents the model adjusted to known confounding factors.

All statistical analyses were performed using the Statistical Package for Social Sciences (SPSS) 18.0, and all statistical tests were two-sided, with differences considered statistically significant at *p* < 0.05.

### Institutional review board statement

This study protocol was approved by the Ethics Committee of the Affiliated Hospital of Zunyi Medical University on December 31, 2021, with the approval number: (KLLY-2021-149).

### Informed consent statement

Informed consent was obtained from all subjects involved in the study.

## Results

### Participants

**Figure 1:**
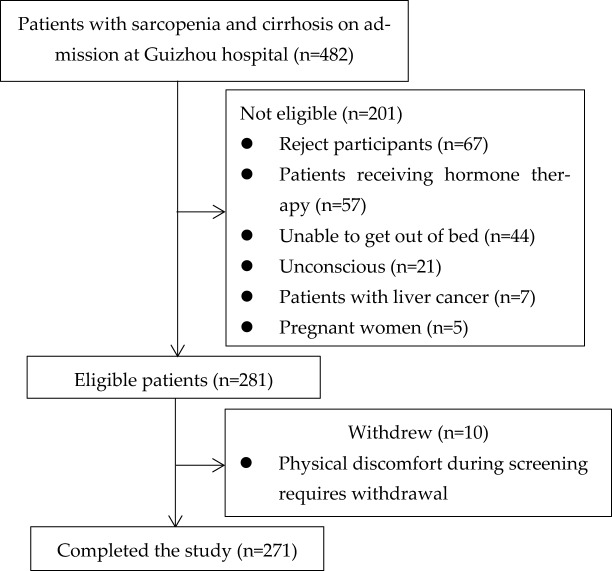
Flow chart showing study participant selection.

A total of 482 hospitalized patients with LC initially met the inclusion criteria for the study and were selected for qualification screening. However, 201 of these patients met the exclusion criteria and were not eligible to participate. Of the eligible 281 cirrhotic patients, 10 of them were withdrawn due to physical discomfort during the screening process. Finally, 271 participants completed the study (Fig. 1).

### Descriptive data

The number of participants with sarcopenia was 75 (27.7%). Notably, 27.3% (50/183) of male participants and 28.4% (25/88) of female participants had sarcopenia (*p* = 0.851). The prevalence of sarcopenia increased with age, with rates of 24.6% for those under 60 years old and 38.3% for those aged 60 or older. The prevalence of sarcopenia was 38.9% (28/72) in alcohol drinkers compared to 23.6% (47/199) in non-drinkers (*p* = 0.013). The prevalence of sarcopenia was related to exercise status: 36.4% and 16.7% for < 3 times/week and ≥ 3 times/week, respectively (*p* < 0.001) (Table 2).

**Table 2:** Comparison of socio-demographic data between the sarcopenic group and the non-sarcopenic group.

Projects	Total (N = 271)	Sarcopenia group (n = 75)	Non-sarcopenia group (n = 196)	*p* value
Gender, n (%)				
Male	183 (67.5)	50 (27.3)	133 (72.7)	0.851
Female	88 (32.5)	25 (28.4)	63 (71.6)	
Age category, n (%)				
< 60	211 (77.9)	52 (24.6)	159 (75.4)	0.037
≥ 60	60 (22.1)	23 (38.3)	37 (61.7)	
Marital status, n (%)				
Married	238 (87.8)	60 (25.2)	178 (74.8)	0.054
Divorced	7 (2.6)	4 (57.1)	3 (42.9)	
Widowed	17 (6.3)	8 (47.1)	9 (52.9)	
Unmarried	9 (3.3)	3 (33.3)	6 (66.7)	
Residential area, n (%)				
Rural	159 (58.7)	47(29.6)	112 (70.4)	0.409
Urban	112 (41.3)	28(25.0)	84 (75.0)	
Co-living status, n (%)				
Living alone	34 (12.5)	10(29.4)	24(70.6)	0.424
Living with family	205 (75.6)	57(27.8)	148(72.2)	
Living with children	20 (7.4)	7(35.0)	13(65.0)	
Living with others	12 (4.4)	1(8.3)	11(91.7)	
Smoking, n (%)				
No	158 (58.3)	38(24.1)	120(75.9)	0.115
Yes	113 (41.7)	37(32.7)	76(67.3)	
Drinking, n (%)				
No	199 (73.5)	47(23.6)	152(76.4)	0.013
Yes	72 (26.5)	28(38.9)	44(61.1)	
Physical activity ≥ 30 min/time, n (%)				
≥ 3 times/week	120 (44.3)	20(16.7)	100(83.3)	< 0.001
< 3 times/week	151 (55.7)	55(36.4)	96(63.6)	
Monthly income, n (%)				
< 1000	50 (18.5)	18(36.0)	32(64.0)	0.219
1000–3000	90 (33.2)	19(21.1)	71(78.9)	
3001–5000	86 (31.7)	23(26.7)	63(73.3)	
> 5000	45 (16.6)	15(33.3)	30(66.7)	
Medical insurance mode, n (%)				
URR medical insurance	240 (88.6)	65(27.1)	175(72.9)	0.544
Employee health insurance	31 (11.4)	10(32.3)	21(67.7)	

*URR* urban–rural resident.

### Outcome data

Compared with participants without sarcopenia, subjects with combined sarcopenia had higher hospital costs, longer hospitalization periods (*p* < 0.001), and were more likely to develop spontaneous bacterial peritonitis, hepatic encephalopathy, ascites, and electrolyte disturbances (*p* < 0.05). Furthermore, the severity of liver function was strongly associated with the development of sarcopenia (*p* = 0.001) (Table 3).

**Table 3:** Comparison of disease-related information between the sarcopenic group and the non-sarcopenic group.

Projects	Total (N = 271)	Sarcopenia group (n = 75)	Non-sarcopenia group (n = 196)	*p* value
Etiology of cirrhosis, n (%)				
Hepatitis B	155 (57.2)	35 (22.6)	120(77.4)	0.247
Hepatitis C	15 (5.5)	5 (33.3)	10(66.7)	
Alcoholic hepatitis	29 (10.7)	10 (34.5)	19(65.5)	
Combined	26 (9.6)	8 (30.8)	18(69.2)	
Others	46 (17.0)	17(37.0)	29(63.0)	
Duration of cirrhosis, n (%)				
< 1 year	108 (39.9)	27 (25.0)	81(75.0)	0.843
1–5 years	64 (23.6)	20 (31.3)	44(68.7)	
6–10 years	38 (14.0)	11(28.9)	27(71.1)	
> 10 years	61 (22.5)	17(27.9)	44(72.1)	
Stage of cirrhosis, n (%)				
Compensatory	45 (16.6)	9(20.0)	36(80.0)	0.208
Decompensation	226 (83.4)	66(29.2)	160(70.8)	
Hospitalization, median (IQR))				
Days	12 (11)	14(10)	10(11.75)	< 0.001
Cost, thousands of RMB	11.49 (13.46)	15.02 (9.11)	9.66 (11.30)	< 0.001
Complications, n (%)				
Hypersplenism	63(23.2)	23 (36.5)	40 (63.5)	0.074
SBP	77 (28.4)	31 (40.3)	46 (59.7)	0.004
HE	77 (28.4)	34 (44.2)	43 (55.8)	< 0.001
Ascites	84 (31.0)	31 (36.9)	53 (63.1)	0.023
HRS	32 (11.8)	13 (40.6)	19 (59.4)	0.081
Electrolyte disorders	34 (12.5)	15 (44.1)	19 (55.9)	0.022
Comorbidities, n (%)				
Diabetes mellitus	44 (16.2)	14 (31.8)	30 (68.2)	0.502
Hypertension	42 (15.5)	14(33.3)	28(66.7)	0.373
Child–Pugh classification, n (%)				
A + B	187 (69.0)	40 (21.4)	147 (78.6)	0.001
C	84 (31.0)	35(41.7)	49(58.3)	

*IQR*, interquartile range, *HE* hepatic encephalopathy, *HRS* hepatorenal syndrome, *SBP* spontaneous bacterial peritonitis.

The median hand grip strength was 22.8 kg for participants with sarcopenia and 28.9 kg for those without sarcopenia. Participants with sarcopenia had lower L3-SMI, L3-SMA, gait speed, total cholesterol, albumin, weight, BMI, and mid-arm muscle circumference than those without comorbidities (*p* < 0.001). Additionally, participants with sarcopenia had higher total bilirubin than those without sarcopenia (*p* < 0.001) (Tables 4&5). Participants with sarcopenia had lower hemoglobin, triglycerides, high-density lipoprotein, and TSF than those without comorbid sarcopenia (*p* < 0.05). Furthermore, participants with sarcopenia had higher gamma-glutamyl transferase and alkaline phosphatase than those without sarcopenia (*p* < 0.05). The incidence of sarcopenia in cirrhotic patients with high malnutrition risk was higher than that in patients with low-medium malnutrition (*p* < 0.05) (Table 5).

**Table 4:** Comparison of muscle-related indicators between the sarcopenic group and the non-sarcopenic group.

Projects	Total (N = 271)	Sarcopenia group (n = 75)	Non-sarcopenia group (n = 196)	*p* value
L3-SMI, median (IQR), cm^2^/m^2^	51.16 (13.77)	41.74 (9.78)	53.17 (10.97)	< 0.001
L3-SMA, median (IQR), cm^2^	128.94 (47)	103.18 (39.1)	136.49 (45.88)	< 0.001
HGS, median (IQR), kg	26.5 (13.8)	22.8 (9.7)	28.9 (13.85)	< 0.001
Gait speed, median (IQR), m/s	1.06 (0.23)	0.86(0.23)	1.12 (0.19)	< 0.001

*L3-SMI* L3 skeletal muscle mass index, *L3-SMA* L3 skeletal muscle area, *HGS* hand grip strength.

**Table 5:** Comparison of laboratory and anthropometric indices between the sarcopenic group and the non-sarcopenic group.

Projects	Total (N = 271)	Sarcopenia group(n = 75)	Non-sarcopenia group (n = 196)	*p* value
Cirrhosis-related biochemical data				
Hemoglobin, mean (SD), g/L	108.33 (27.66)	100.07 (24.07)	111.49 (28.34)	0.001
INR	1.29 (0.36)	1.35 (0.34)	1.27 (0.36)	0.132
Triglycerides, mean (SD), mmol/L	1.34 (0.89)	1.10 (0.56)	1.43 (0.97)	0.007
Cholesterol, mean (SD)				
Total, mmol/L	3.40 (1.06)	2.93 (0.84)	3.58 (1.08)	< 0.001
HDL, mmol/L	0.88 (0.39)	0.80 (0.39)	0.91 (0.39)	0.046
LDL, mmol/L	2.12 (0.79)	2.11 (0.81)	2.12 (0.78)	0.946
FBS, mean (SD), mmol/L	6.71 (3.64)	7.36 (4.26)	6.46 (3.35)	0.068
Liver functions, median (IQR)				
ALT, UL	48 (132)	59 (124)	47 (132.75)	0.99
AST, U/L	80 (126)	102 (158)	75 (117.5)	0.284
GGT, U/L	72 (116)	94 (156)	68 (111.5)	0.012
ALP, U/L	137 (91)	152 (117)	133.5 (82.25)	0.011
TBil, μmol/L	48.7 (93.6)	75.5 (158.9)	36.4 (75.28)	< 0.001
Total protein, mean (SD), g/L	63.55 (9.81)	63.68 (12.27)	63.50 (8.72)	0.892
Albumin, mean (SD), g/L	31.13 (5.69)	27.20 (4.26)	32.64 (5.45)	< 0.001
Anthropometric data				
Height, mean (SD), cm	159.52 (8.47)	158.26 (8.04)	160.01 (8.6)	0.13
Weight, mean (SD), kg	58.72 (9.32)	52.20 (6.35)	61.21 (9.07)	< 0.001
BMI, mean (SD), kg/m^2^	23.01 (3.17)	20.83 (1.92)	23.84 (3.17)	< 0.001
TSF, mean (SD), cm	10.49 (4.89)	9.27 (4.34)	10.95 (5.01)	0.011
MAMC, mean (SD), cm	22.99 (3.19)	21.11 (2.55)	23.72 (3.11)	< 0.001
WHR, mean (SD), cm	0.96 (0.10)	0.94 (0.08)	0.96 (0.10)	0.069
Risk of malnutrition, n (%)				
Low-medium risk of malnutrition	130 (48.0)	24 (32.0)	106 (54.1)	0.001
High risk of malnutrition	141 (52.0)	51 (68.0)	90 (45.9)	

*SD* standard deviation, *INR* international normalized ratio, *FBS* fasting blood sugar, *ALT* glutamic-pyruvic transaminase, *AST* glutamic-oxalacetic transaminase, *GGT* gamma-glutamyl transferase, *ALP* alkaline phosphatase, *TBil* total bilirubin, *BMI* body mass index, *TSF* triceps skinfold thickness, *MAMC* mid-upper arm muscle circumference, *WHR* waist-to-hip ratio.

### Outcome data

The binary ordered regression with sarcopenia as the dependent variable revealed several significant associations. In the adjusted model for the associated risk factors for sarcopenia (Model 2), the prevalence of sarcopenia increased with decreasing physical activity frequency (OR 2.498; 95% CI 1.063–5.874). High malnutrition risk was also linked to a higher prevalence of sarcopenia (OR 2.579; 95% CI 1.127–5.898), as was hepatic encephalopathy (OR 3.658; 95% CI 1.539–8.694). Age (OR 2.688; 95% CI 1.012–7.137) and high ALP levels (OR 1.007; 95% CI 1.003–1.011) were associated with a high risk of sarcopenia. In contrast, BMI (OR 0.663; 95% CI 0.551–0.799), MAMC (OR 0.833; 95% CI 0.716–0.969), albumin (OR 0.831; 95% CI 0.762–0.907), and total cholesterol (OR 0.389; 95% CI 0.230–0.659) were negatively correlated with sarcopenia risk. After further adjustment for gender in Model 3, the association between age and risk of sarcopenia was reduced (OR 2.631; 95% CI 0.981–7.058) (Table 6).

**Table 6:** Logistic regression analysis of factors influencing the development of sarcopenia in patients with liver cirrhosis.

Variables	Model 1			Model 2 (R^2^ = 0.653)			Model 3 (R^2^ = 0.653)		
	OR	95% CI	*p* value	OR	95% CI	*p* value	OR	95% CI	*p* value
Age (year)									
< 60 (ref)	1			1			1		
≥ 60	1.901	1.035–3.489	0.038	2.688	1.012–7.137	0.047	2.631	0.981–7.058	0.055
Physical activity ≥ 30 min/time									
≥ 3 times/week (ref)	1			1			1		
< 3 times/week	2.865	1.598–5.134	< 0.001	2.498	1.063–5.874	0.036	2.530	1.069–5.989	0.035
BMI, kg/m^2^	0.623	0.538–0.721	< 0.001	0.663	0.551–0.799	< 0.001	0.665	0.552–0.801	< 0.001
MAMC (cm)	0.741	0.666–0.824	< 0.001	0.833	0.716–0.969	0.018	0.826	0.704–0.970	0.020
Hepatic encephalopathy									
No (ref)	1			1			1		
Yes	2.951	1.674–5.200	< 0.001	3.658	1.539–8.694	0.003	3.623	1.524–8.614	0.004
Nutritional status									
Low-medium malnutrition risk (ref)	1			1			1		
High risk of malnutrition	2.503	1.429–4.384	0.001	2.579	1.127–5.898	0.025	2.545	1.108–5.844	0.028
ALP (U/L)	1.004	1.001–1.007	0.003	1.007	1.003–1.011	< 0.001	1.007	1.003–1.011	< 0.001
Albumin (g/L)	0.810	0.760–0.863	< 0.001	0.831	0.762–0.907	< 0.001	0.832	0.762–0.908	< 0.001
Total cholesterol (mmol/L)	0.473	0.339–0.659	< 0.001	0.389	0.230–0.659	< 0.001	0.389	0.229–0.659	< 0.001
Gender									
Male (ref)	1			–	–	–	1		
Female	1.056	0.599–1.859	0.851	–	–	–	0.870	0.338–2.239	0.772

Model 1 included exercise, BMI, MAMC, hepatic encephalopathy, nutritional status, ALP, albumin, and total cholesterol. Model 3 adjusted for gender based on Model 2.

*OR* odds ratio, *CI* confidence interval, *ref* Reference group of the categorical variable.

## Discussion

In this study, hepatitis B infection was identified as the leading cause of cirrhosis, while alcoholic hepatitis was a common disease abroad. The prevalence of sarcopenia was higher in patients with cirrhosis (27.68%). In Model 1, the risk of sarcopenia was positively associated with age, physical activity < 3 times/week, high risk of malnutrition, hepatic encephalopathy, and high ALP levels. Conversely, it was negatively associated with high BMI, MAMC, albumin, and total cholesterol. After adjusting for gender in Model 3, the association between age and the risk of sarcopenia was attenuated. In addition, patients with advanced liver disease had a higher likelihood of developing sarcopenia.

The prevalence of sarcopenia in patients with cirrhosis remains a subject of debate. A study that included 201 subjects with cirrhosis reported a prevalence of sarcopenia 57.2%, with no significant difference between males and females^19^. Kim et al. found that the complication rate of sarcopenia in patients with LC was 30%–70%, with a higher prevalence among males^20^. In the present study, there was no statistical difference in the prevalence of sarcopenia between males and females. This disparity in findings may be attributed to the differences in racial characteristics, body size, and disease etiology between individuals from Asian and Western countries. Notably, the prevalence of sarcopenia has been reported as 10% for Child–Pugh A, 34% for Child–Pugh B, and 54% for Child–Pugh C^21^, similar to the results of the present study, suggesting that muscle mass loss in cirrhotic patients is more pronounced as the liver reserve deteriorates.

The present study made extensive efforts to account for potential confounding variables and identified aging as a significant risk factor for developing sarcopenia. However, this association was attenuated after adjusting for gender. This attenuation may be due to the small sample size of patients in the study. Research has shown that there can be a substantial loss of skeletal muscle mass, estimated at 20–30%, from ages 20 to 80^22^. Muscle strength begins to decline around the age of 30 and declines rapidly around the age of 50 years^23^. These age-related changes are characterized by motor unit remodeling, increased muscle fiber denervation, reduced protein synthesis, and a decrease in the number of muscle satellite cells required for skeletal muscle growth and repair^6^, ultimately leading to a decrease in muscle fiber cross-sectional area. Therefore, both the AWGS 2019 and the European Working Group on Sarcopenia in Older People 2 (EWGSOP 2) recommend early screening and intervention for sarcopenia in older adults to alleviate the burden of this condition in a high-risk population^1,13^.

Cirrhosis is usually associated with protein-energy malnutrition and low physical activity, leading to sarcopenia, with the prevalence of protein malnutrition ranging from 20 to 30% in patients with chronic liver disease (CLD) to over 60% in patients with cirrhosis^24^. Sarcopenia, characterized by decreased muscle/fat mass, increased pro-inflammatory cytokines, and anorexia, can potentially be mitigated with nutritional supplements, potentially slowing its progression to sarcopenia^25^. In the present study, a high risk of malnutrition (OR 2.579; 95% CI, 1.127–5.898) was associated with an increased risk of sarcopenia in cirrhotic patients with physical activity < 3 times/week (OR 2.498; 95% CI, 1.063–5.874). High ALP levels were also associated with the development of sarcopenia in cirrhotic patients. Lee et al.^26^ explored the relationship between serum ALP levels and low muscle mass index in 15,579 adults in Korea, and the findings suggested that serum ALP may serve as a marker of inflammation and a predictor of sarcopenia. Further research is needed to confirm whether ALP can predict sarcopenia development in cirrhosis patients. Serum albumin levels have been recognized as an important factor associated with decreased muscle size and strength^27^. Montano et al.^28^ also confirmed that decreased albumin levels were independently associated with a higher risk of sarcopenia and a poor prognosis. In the present study, albumin levels were significantly associated with the risk of sarcopenia in patients with cirrhosis. Regression analysis showed that normal total cholesterol levels were associated with the development of sarcopenia in cirrhotic patients. Previous studies have reported that obesity due to abnormal total cholesterol levels is a significant risk factor for the development of sarcopenia in cirrhotic patients^29^.

BMI is an indicator to assess the nutritional status of the body. A low BMI typically indicates low body fat. Several studies have confirmed that L3-SMI is positively correlated with BMI^30,31^. In our study, the median BMI among participants with sarcopenia was 22.8 kg/m^2^, consistent with the findings of Landi^32^, who found that older people with a BMI greater than 21 kg/m^2^ had 0.76 times the risk of sarcopenia compared to those with a BMI less than 21 kg/m^2^. MAMC is strongly associated with lean muscle mass and body fat, has good intra- and inter-observer reproducibility, and has been shown to predict mortality^33,34^. While muscle mass is crucial, muscle strength is a more significant factor in determining physical function^35^. HGS has proven to be a better predictor of poor clinical outcomes than CT measurements of muscle mass and the Model for End-Stage Liver Disease. Even small improvements in HGS, as little as 1 kg, suggest a significant reduction in mortality^36,37^. Early identification of sarcopenia through routine imaging, convenient assessment tools, and clinical observation is an integral part of the care of patients with cirrhosis. Considering that screening tests need to be of high sensitivity, we recommend the combination of MAMC and grip strength as an initial screening test for sarcopenia rather than SARC-F in patients with CLD. SARC-F has demonstrated low sensitivity (15%–45%) in patients with CLD^38^, limiting its clinical application. Early detection of sarcopenia facilitates timely referral for confirmation of the diagnosis and implementation of primary prevention measures in high-risk populations.

This study confirms that hepatic encephalopathy is an independent risk factor for the development of sarcopenia. Hyperammonemia has been shown to increase muscle growth inhibitor expression through toll-like receptor—independent activation of nuclear factor kappa β in animal models^39^, increasing the risk of developing sarcopenia in patients with cirrhosis. Furthermore, Nardelli et al.^40^ reported an association between the development of sarcopenia in patients with cirrhosis and the development of hepatic encephalopathy. Several studies have reported that branched-chain amino acids (BCAA) are beneficial in the treatment of hepatic encephalopathy^41^. For cirrhotic patients with protein intolerance or inability to meet protein targets, guidelines recommend a BCAA intake of 0.25 g·kg^−1^d^−1^^42^. However, there is a paucity of research on the potential of BCAA in ameliorating sarcopenia.

The current study has several limitations. First, this is a case–control study conducted in a local hospital in China, which could potentially limit the generalizability of the findings. Second, there was no healthy control group to serve as a reference for comparing the prevalence of sarcopenia; therefore, only indirect comparisons could be made. Third, the sample size was relatively small, and there may be inherent bias among cases. In future research, we plan to address these limitations by including a larger cohort of cirrhotic patients and conducting follow-up assessments to enhance the study's reliability and generalizability.

## Conclusions

In conclusion, this study found a higher prevalence of sarcopenia in patients with cirrhosis. Advanced age, low physical activity, low BMI, decreased MAMC, hepatic encephalopathy, and nutritional status were associated with an increased risk of sarcopenia, whereas liver function-related indices such as ALP, albumin, and total cholesterol levels were associated with an increased risk of sarcopenia. These findings suggest that promoting physical activity in cirrhotic patients, with a focus on reducing body fat and enhancing cognitive function, plays a crucial role in preventing sarcopenia.

## Data Availability

Data associated with this study can be obtained by reasonable request to the corresponding author (P.Y.).
