# Author Correction: Assessing the risk factors associated with sarcopenia in patients with liver cirrhosis: a case–control study

**DOI:** 10.1038/s41598-024-80824-1

**Published:** 2024-12-10

**Authors:** LeYao Xiao, Mei Dai, Fei Zhao, YouShu Shen, Rick Yiu Cho KWAN, Jordan Tovera Salvador, Li Zhang, YaWen Luo, Qian Liu, Ping Yang

**Affiliations:** 1 Department of Infectious Diseases, Affiliated Hospital of Zunyi Medical University, Zunyi, 563000 China; 2 School of Nursing, Zunyi Medical University, Zunyi, 563000 China; 3 School of Nursing, Tung Wah College, Hong Kong SAR, China; 4 Nursing Education Department, College of Nursing, Imam Abdulrahman Bin Faisal University, Dammam, Saudi Arabia; 5 Nursing Department of the Affiliated Hospital of Zunyi Medical University, Zunyi, China; 6 Philippine Women’s University, Manila, Philippines

Correction to: *Scientific Reports* 10.1038/s41598-023-48955-z, published online 09 December 2023.

In the original version of this Article Jordan Tovera Salvador was incorrectly affiliated with ‘Philippine Women’s University, Manila, Philippines’. The correct affiliation is listed below:

Nursing Education Department, College of Nursing, Imam Abdulrahman Bin Faisal University, Dammam, Saudi Arabia.

As a result, the Affiliations have been renumbered.

The original Article has been corrected.

